# MicroRNA-1247 inhibits cell proliferation by directly targeting ZNF346 in childhood neuroblastoma

**DOI:** 10.1186/s40659-018-0162-y

**Published:** 2018-05-24

**Authors:** Tingting Wu, Yun Lin, Zhongguo Xie

**Affiliations:** 1 Department of Neonatology, The First People’s Hospital of Jingzhou, No. 8 Hangkong Road, Shashi District, Jingzhou, 434000 Hubei China; 2 Department of Editor, Sichuan Academy of Medical Sciences and Sichuan Provincial People’s Hospital, Sichuan, China

**Keywords:** Neuroblastoma, miR-1247, Proliferation, Apoptosis, ZNF346

## Abstract

**Background:**

Neuroblastoma (NB) represents the most common extracranial solid tumor in children. Accumulating evidence shows that microRNAs (miRs) play an important role in the carcinogenesis of NB. Here, we investigated the biological function of miR-1247 in NB in vitro.

**Methods/results:**

We found miR-1247 was downregulated in NB tissues and cells using quantitative PCR analysis. Gain- and loss-of-function studies demonstrated that miR-1247 significantly suppressed cell proliferation and induced cell cycle G0/G1 phase arrest and cell apoptosis of NB cells in vitro by using MTT, colony formation assay and Flow cytometry analysis. Luciferase assay suggested ZNF346 was the target of miR-1247 and its expression could be downregulated by miR-1247 overexpression using Western blotting. Furthermore, downregulation of ZNF346 by siRNA performed similar effects with overexpression of miR-1247 in NB cells.

**Conclusions:**

Our findings suggested miR-1247 directly targeted to repress ZNF346 expression, thus suppressing the progression of NB, which might be a novel therapeutic target against NB.

## Introduction

Neuroblastoma (NB) is a malignancy of peripheral sympathetic nervous system that frequently occurs in children and is derived from the improper differentiation of primitive neural crest cells during embryonic development [1, 2]. These tumors usually arise in the adrenal medullar, posterior mediastinum, and abdominal sympathetic ganglia [3, 4]. Although NB constitutes only approximately 8% of pediatric malignancies, it is responsible for 15% of cancer-related deaths [5]. In recent years, remarkable therapeutic management has been made for children with early stage NB. However, patients with advanced stages remain practically incurable despite dramatic escalations in the cytotoxic chemoradiotherapy. Hence, there is an urgent medical need for exploration of new treatment strategies.

MicroRNAs (miRs) are a class of small, ~ 22- nucleotide long non-coding RNAs, which completely or partially complementary with the 3′UTR of their target mRNA to directly repress post-transcription [6, 7]. In fact, a surprisingly large number of annotated miRs (with more than 1000) have been found in humans and considered to affect multiple physiological and developmental processes [8]. Moreover, accumulating reports have described dysfunction and implication of miRs in various diseases, including cancer, cardiovascular and Alzheimer’s disease [9–13]. Recently, several tumorigenic suppression or promotion property procedures, miRNAs, such as miR-584-5p targeting matrix metalloproteinase 14 [14], miR-329 and miR-137 targeting KDM1A [15, 16], miR-542-3p downregulating Survivin expression [17], and miR-92a repressing TrkA expression [18], have been shown in NB.

One miRNA that has obtained special attention in the cancer research field is microRNA-1247 (miR-1247), the expression of which is altered in pancreatic cancer [19], prostate cancer [20], non-small-cell lung cancer (NSCLC) [21] and hepatocellular carcinoma [22]. Shi et al. [19] showed that high expression of miR-1247 related to prognosis of pancreatic cancer and depressed scell growth through targeting neuropilins. DNA methylation induced knockdown of miR-1247 enhanced proliferation and metastasis of NSCLC cells [21]. Furthermore, miR-1247 exerted antitumor effects on hepatocellular carcinoma by inhibition proliferative and invasive capabilities of tumor cells [22]. Whereas, the role and mechanism of miR-1247 in NB has never been reported. ZNF346, which is widely expressed in brain, belongs to a new class of C2H2-type zinc finger proteins [23]. It is also a SIRT-interacting protein that protects neurons in tissue culture models of neurodegenerative disease by suppressing cell-cycle re-entry though triggering expression of p21 [24]. In the present study, we have investigated miR-1247 expression patterns in NB and adjacent tissues. Subsequently, we further investigated the roles of miR-1247 in the regulation of the malignant phenotypes of NB and its underlying mechanisms.

## Materials and methods

### Patient tissue samples

Fresh tumor specimens from 10 well-established primary NB cases and the corresponding adjacent normal nerve tissues were collected from the Department of Pediatrics, the First People’s Hospital of Jingzhou (Hubei, China) and snap frozen immediately at − 80 °C until use. The pathological diagnosis of NB was confirmed by at least two pathologists. These 10 patients include 4 female and 6 male. The mean age is 45 years. According to international neuroblastoma staging system, 4 patients were classified as stage 1, 4 as stage 2 and 3 as stage 3. Patient’s written consent and approval to conduct this study was obtained from the First People’s Hospital of Jingzhou.

### Cell culture and transfection

Human NB cell lines (SH-SY5Y and SK-N-SH) were purchased from Shanghai Institute of Biochemistry and Cell Biology (Shanghai, China) and cultured in RPMI-1640 medium supplemented with 10% FBS, 100 IU/ml penicillin and 10 mg/mL streptomycin. All cells were maintained in an atmosphere of 5% CO_2_ at 37 °C.

For cell transfection, the miR-1247 mimics, miR-1247 inhibitor, small interfering RNA for ZNF346 (siZNF346) and their corresponding negative control oligonucleotides (NC-mimics, NC-inhibitor and si-NC, respectively) were chemically synthesized by GenePharma Co. Ltd. (Shanghai, China). The 100 nmol of above oligonucleotides were transfected into confluent cells with Lipofectamine 2000 (Invitrogen, Carlsbad, CA, USA) according to the manufacture’s instruction. After 48 h of transfection, cells were used for the following in vitro experiments.

### Quantification of miR-1247 expression

Total miRNA was extracted from primary tissues and cell lines using miRNeasy Mini Kit (Qiagen). Then cDNA was synthesized with a miRNA-specific stem-loop primer and the TaqMan MiRNA Reverse Transcription Kit (Applied Biosystems). Subsequently, the quantitative PCR was performed on a CFX96 Touch Real-Time PCR Detection System (BioRad) using the follow primers: miR-1247 forward: AACGCTCAGCACCCATTTAC and miR-1247 reverse: CGGACGTTGCTCTCTACCC; U6 forward: CTCGCTTCGGCAGCACA and U6 reverse: AACGCTTCACGAATTTGCGT. PCR parameters were as follows: 95 °C for 3 min, 40 cycles of 95 °C for 10 s, and 58 °C for 30 s. Relative miR-1247 expression was calculated using the 2^−ΔΔCt^ method with normalization to U6 snRNA.

### Western blot

Transfected cells were seeded at a density of 1 × 10^6^ cells per dish in 6-cm dishes. After 96 h of culture, cellular protein was extracted with RIPA buffer (20 mM Tris–HCl pH 7.5, 10 mM EDTA, 150 mM NaCl, and 1% Sodium dexoycholate, 0.1% SDS). Protein concentration was determined using a BCA protein assay kit (Thermo). Following denaturation in boiling water for 5 min, approximately 30 µg of protein extracts were separated on 10% SDS-PAGE gels and transfected onto polyvinylidene fluoride (PVDF) membranes (Millipore, MA, USA). After blocked with non-fat milk, the membranes were incubated with primary antibodies against ZNF346 (1:1000, #35964, Cell Signaling Technology), CDK1 (1:1000, 11026-1-AP, Proteintech), Cyclin D1 (1:1000, 60186-1-1 g, Proteintech), caspase-3 (1:1000, 25546-1-AP, Proteintech), Bcl-2 (1:1000, #2876, Proteintech) and GAPDH (1:5000, 10494-1-AP, Proteinintech) overnight at 4 °C and then incubated in the corresponding secondary antibody for 2 h at room temperature. Bands were detected with Enhanced chemiluminescence substrate kit (Amersham, Piscataway, NJ). GAPDH was used as an internal control.

### MTT assay

Cells were planted in 96-well plates at a density of 3,000 cells per well. A total of 100 µl of MTT solution (0.5 mg/ml, Sigma-Aldrich, St. Louis, MO, USA) was added into each well and the cells were incubated for 4 h at 37 °C. At 1, 2, 3, 4, and 5 day after culture, the optical density (OD) value in each well was recorded using a microplate reader at 595 nm wavelength.

### Colony formation

Cells with miR-1247 overexpression or knockdown were seeded into 6-well plates at a density of 500 cells per well. After cultured for 5 days, the cells were washed with PBS and fixed with 10% formaldehyde for 5 min followed by staining with 0.5% crystal violet for 3 min. The colonies (more than 50 cells per colony) was photographed and manually counted with an inverted microscope.

### Flow cytometry analysis

Cell cycle distribution was detected using propidium iodide (PI) staining. Briefly, transfected cells were seeded at a density of 1 × 10^6^ cells per dish in 6-cm dishes. After 48 h of incubation, cells were collected and fixed in pre-cooling 70% ethanol at 4 °C overnight. The following day, immobilized cells were collected, wished and resuspended in 500 μl PBS containing 50 μg/ml PI, 100 μg/ml RNase A and 0.2% Triton X-100. After incubated in dark at room temperature for 30 min, the cells were analyzed using a BD FACSCalibur Flow Cytometer (BD Biosciences, CA, USA).

Cells apoptosis was analyzed using AnnexinV-FITC/PI Kit (BD Pharminogen, USA) according to the manufacturer’s instructions. Briefly, transfected cells were seeded in 6-cm dishes at a density of 1 × 10^6^ cells per dish. After 48 h of culture, the cells were collected and stained with Annexin V-FITC (5 μl) and PI (10 μl) in the dark. After washing twice with PBS, the cells were collected and analyzed using a BD FACSCalibur Flow Cytometer (BD Biosciences, CA, USA). Each treatment was performed for three times.

### Dual-luciferase activity assay

The interaction between 3′-UTR of ZNF346 and miR-1247 was detected using Luciferase activity assay. The psiCHECKTM vector used for LUC assays was purchased from Promega (Madison, Wisconsin, USA). The wild or mutant type 3′UTR sequences of ZNF346 containing the predicted miR-1247 binding sites were cloned into the psiCHECK-2 vector (Promega Corporation) to construct dual luciferase reporter vector. QuikChangeTM Site-Directed Mutagenesis kit (Agilent Technologies, Inc., Santa Clara, CA, USA) was used to mutagenize the miR-ZNF binding site according to the manufacturer’s protocol. For cell transfection, NB cells were seeded in 96-wells plates and incubated for 24 h. Then cells were harvested and co-transfected with 200 ng of psiCHECK-ZNF346 3′UTR wild type (WT) or psiCHECK-ZNF346 3′UTR mutant (MUT), together with 100 nmol of miR-1247 mimics or NC-mimics using Lipofectamine 2000 (Invitrogen). Forty-eight hours later, cells were measured using the Dual-Luciferase assay system (Promega, Madison, WI, USA) in accordance with the manufacturer’s instructions. Firefly luciferase activity was normalized to Renilla luciferase activity. Each treatment was performed for three times.

### Statistical analysis

Quantitative data are presented as mean ± SD from at least three independent experiments. All statistical analyses were performed using the IBM SPSS Statistics software 21.0. Multiple group comparisons were performed using one-way analysis of variance (ANOVA). The differences between subgroups were analyzed by two-sample Student’s t-tests. A *p* value < 0.05 was considered significant.

## Results

### MiR-1247 was under-expressed in NB tissues

**Fig. 1:**
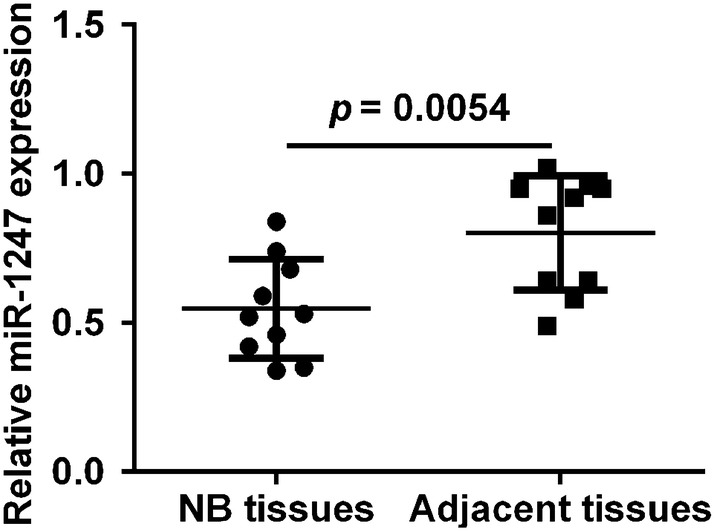
MiR-1247 was downregulated in NB tissues. Quantitative PCR analysis of miR-1247 expression in 10 pairs of NB tumor tissues and adjacent normal tissues

As first step of the study, we conducted quantitative PCR analysis to investigate the expression of miR-1247 in 10 pairs of NB primary tumor tissues and adjacent normal tissues. The result revealed that miR-1247 expression was significantly lower in NB tissues compared with that in adjacent nerve tissues (Fig. 1, *p* = 0.0054), which suggest that miR-1247 might play an important role in the development of nervous system.

### MiR-1247 was associated with cellular proliferative inhibition in NB

**Fig. 2:**
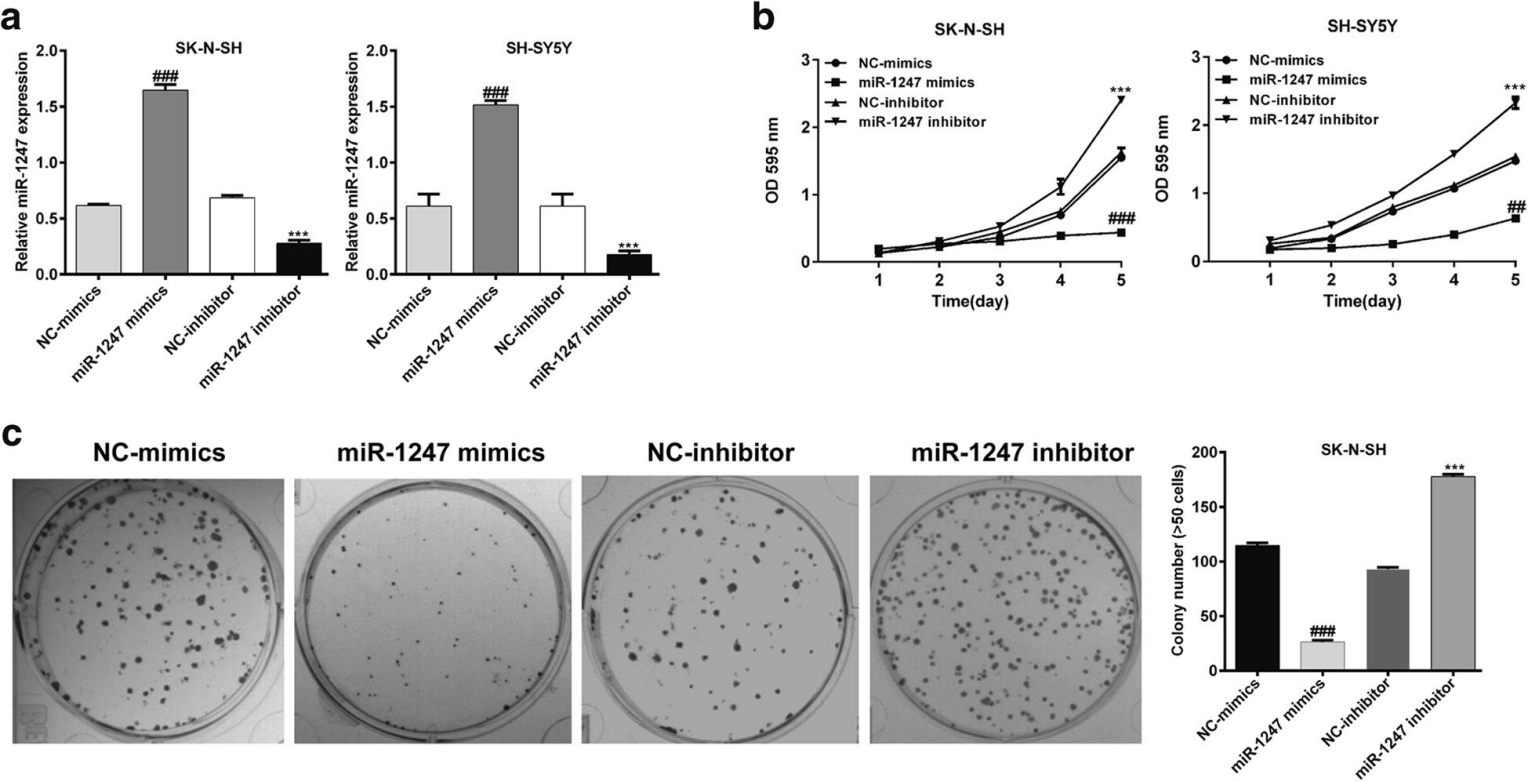
The effects of miR-1247 expression on the proliferation of NB cells. SH-SY5Y and SK-N-SH cells were transfected with the miR-1247 mimics, NC-mimics, miR-1247 inhibitor, or NC-inhibitor, respectively. **a** Quantitative PCR analysis of miR-1247 expression in SH-SY5Y and SK-N-SH cells after 48 h transfection. **b** MTT assay was performed to analyze cell proliferation in SH-SY5Y and SK-N-SH cells after 48 h transfection. **c** The proliferation capacity of SK-N-SH cells was determined by colony formation assay after 48 h transfection. ^##^*p* < 0.01, ^###^*p* < 0.001 versus NC-mimics; ***p < 0.001 versus NC-inhibitor

To explore the possible function of miR-1247 in NB, we used SH-SY5Y and SK-N-SH cells as in vitro cell lines model to detect the effects of miR-1247 expression on the proliferation of neuroblastoma. Firstly, miR-1247 mimics and miR-1247 inhibitor were transfected into these two cell lines to overexpress or reduce miR-1247 expression, respectively. As illustrated in Fig. 2a, the expression of miR-1247 was significantly enhanced in cells transfected with miR-1247 mimics compared with that in NC-mimics, while miR-1247 expression was obviously decreased in cells transfected with miR-1247 inhibitor compared with that in NC-inhibitor (*p* < 0.001). Next, cell proliferation was measured by MTT assay. As shown in Fig. 2b, the cell proliferative rate of NB cells was significantly repressed after transfected with miR-1247 mimics, but elevated after transfected with miR-1247 inhibitor (*p* < 0.01, *p* < 0.01). Furthermore, the proliferative capacity of SK-N-SH cells was further determined by colony formation assay (Fig. 2c). Statistical analysis indicated that colonies formed in cells transfected with miR-1247 mimics was decreased approximately 75.32% compared with that in NC-mimics transfection, while miR-1247 inhibitor transfection remarkably increased the colonies by nearly 89.65%. All of these results demonstrated that miR-1247 might be closely associated with cellular proliferative inhibition in NB.

### MiR-1247 overexpression blocked cell cycle progression in NB cells

**Fig. 3:**
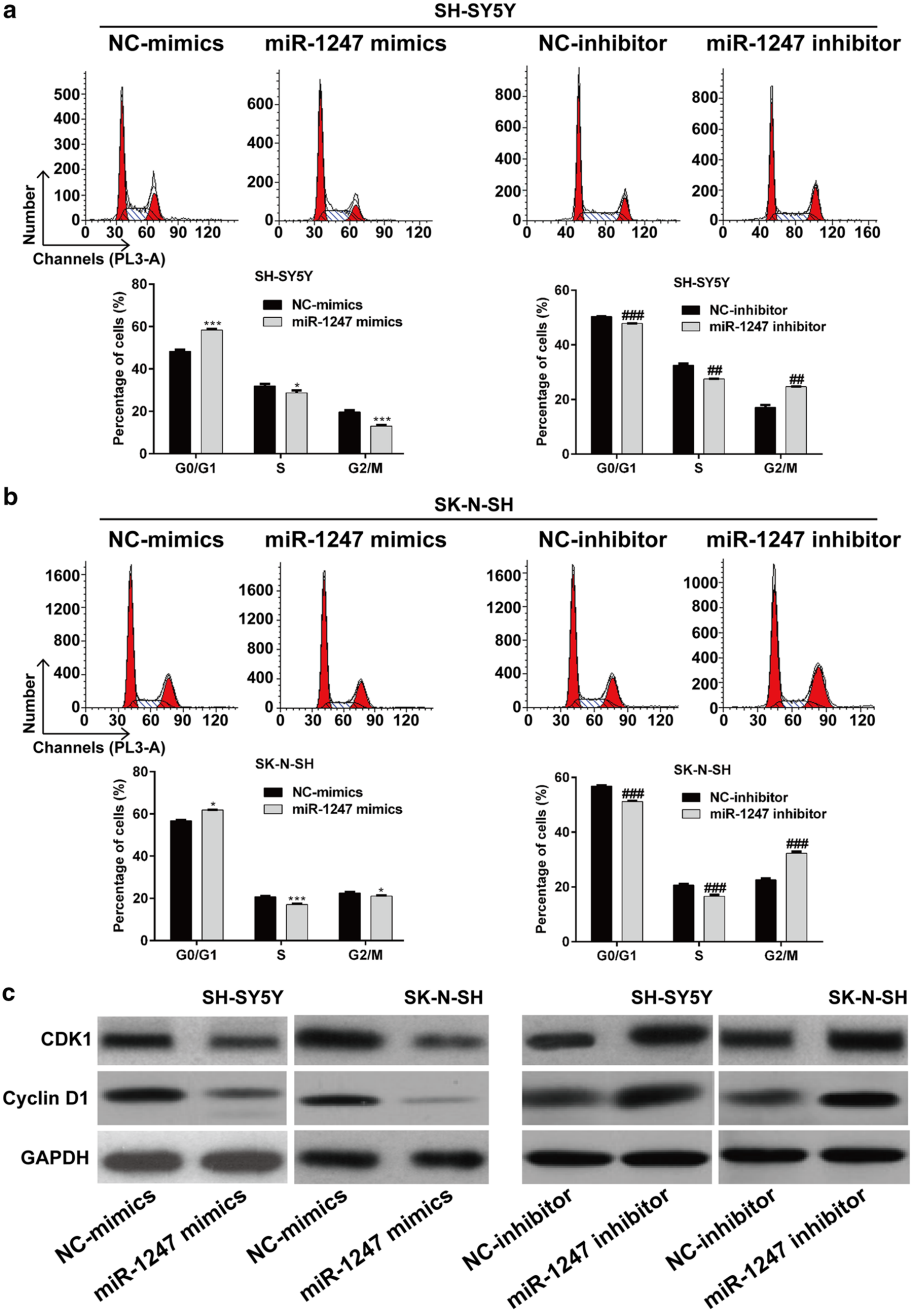
Cell cycle progression in SH-SY5Y and SK-N-SH cells was detected by flow cytometry. **a**, **b** Cells were respectively transfected with the miR-1247 mimics, NC-mimics, miR-1247 inhibitor, or NC-inhibitor, respectively for 48 h. Then the cells were stained with PI and subjected to flow cytometry analysis. The percentage of cells in G0/G1 phase, S phase and G2/M phase was the average value of three repeated experiments. **c** The protein expression of CDK1 and Cyclin D1 were analyzed in SH-SY5Y and SK-N-SH cells infected with miR-1247 mimics, NC-mimics, miR-1247 inhibitor, or NC-inhibitor by Western blot. GAPDH was used as the endogenous control. *p < 0.05, ***p < 0.001 versus NC-mimics; ^##^*p* < 0.01, ^###^*p* < 0.001 versus NC-inhibitor

To further investigate the role of miR-1247 in regulating the proliferation of NB cells, we determined the effects of miR-1247 in regulating cell cycle progression. As shown in Fig. 3a, the cell cycle distribution in NC-mimics group differed from miR-1247 mimics group, and in NC-inhibitor group also differed from miR-1247 inhibitor group in SH-SY5Y cells. Further analysis demonstrated that the percentage of cells in G0/G1 phase was significantly increased (*p* < 0.001), while cells in S and G2/M phase was significantly decreased in miR-1247 mimics group compared with those in NC-mimics group (*p* < 0.05, *p* < 0.001). On the contrary, miR-1247 inhibitor transfection remarkably reduced the percentage of cells in G0/G1 phase, while significantly elevated the percentage of cells in G2/M phase in SH-SY5Y cells (*p* < 0.01, *p* < 0.001). Similarly, miR-1247 overexpression induced cell cycle G0/G1 phase arrest, which could be reversed by miR-1247 knockdown in SK-N-SH cells (Fig. 3b, *p* < 0.05, *p* < 0.001). Furthermore, we detected the expression alterations of some cell cycle regulators. As shown in Fig. 3c, the expression levels of CDK1 and Cyclin D1, associated with G0/G1 phase arrest, were decreased in miR-1247 mimics group compared with those in NC-mimics group, but increased in miR-1247 inhibitor group compared with those in NC-inhibitor group. Collectively, these results further suggested that miR-1247 overexpression could suppress NB cell proliferation might partially through cell cycle arrest.

### MiR-1247 overexpression promoted NB cell apoptosis

**Fig. 4:**
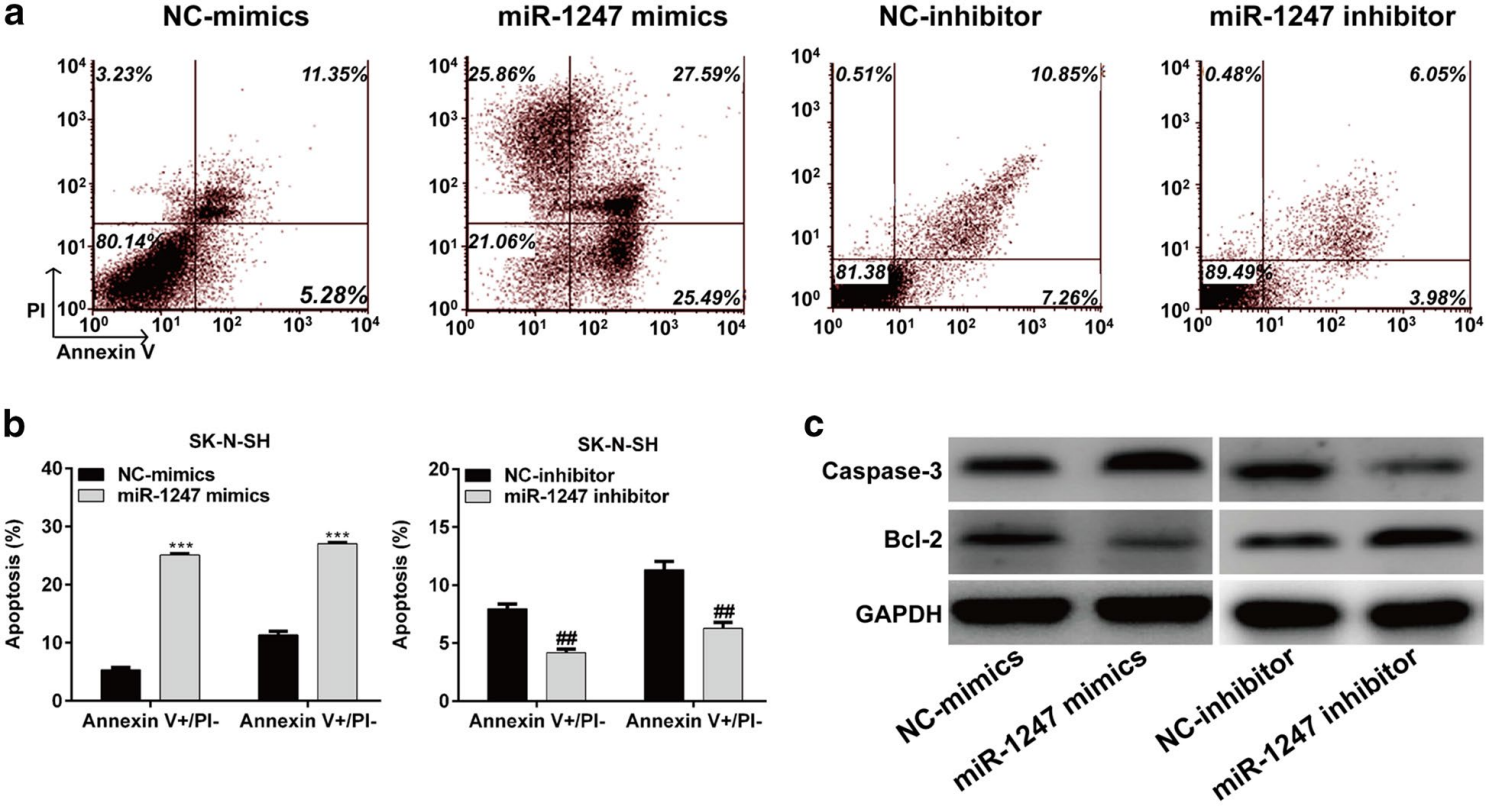
Apoptosis in SK-N-SH was detected by flow cytometry. **a**, **b** Cells were respectively transfected with the miR-1247 mimics, NC-mimics, miR-1247 inhibitor, or NC-inhibitor, respectively for 48 h. Then the cells were stained with Annexin V/PI and subjected to flow cytometry analysis. The percentage cells in early apoptosis (Annexin V+/PI −) and late apoptosis (Annexin V+/PI +) was the average value of three repeated experiments. **c** The protein expression of Caspase 3 and Bcl-2 were analyzed in SK-N-SH cells infected with miR-1247 mimics, NC-mimics, miR-1247 inhibitor, or NC-inhibitor by Western blot. GAPDH was used as the endogenous control. ^##^*p* < 0.01 versus NC-inhibitor; ***p < 0.001 versus NC-mimics

Subsequently, we determined the apoptosis in SK-N-SH cells using flow cytometry analysis. As shown in Fig. 4a, b, the percentage of cells in early apoptosis (Annexin V+/PI−) were about 25.08% or 5.36% transfected with miR-1247 mimics or NC-mimics, respectively in SK-N-SH cells. Similarly, the percentage of cells in late apoptosis (Annexin V+/PI+) was significantly increased from 11.36% in miR-1247 mimics group to 27.04% in NC-mimics in SK-N-SH cells (*p* < 0.001). On the contrary, miR-1247 inhibitor transfection notably reduced the early and late apoptotic cells in SK-N-SH cells (*p* < 0.01). Moreover, we detected the expression alterations of some apoptotic markers. As shown in Fig. 4c, the expression of caspase-3 was increased, while the anti-apoptotic protein Bcl-2 was decreased in SK-N-SH cells following miR-1247 mimics transfection. However, miR-1247 knockdown remarkably reduced caspase-3 expression and elevated Bcl-2 expression in SK-N-SH cells. These results further demonstrated that miR-1247 overexpression could promote NB cell apoptosis.

### ZNF346 was a target gene of miR-1247 in NB

**Fig. 5:**
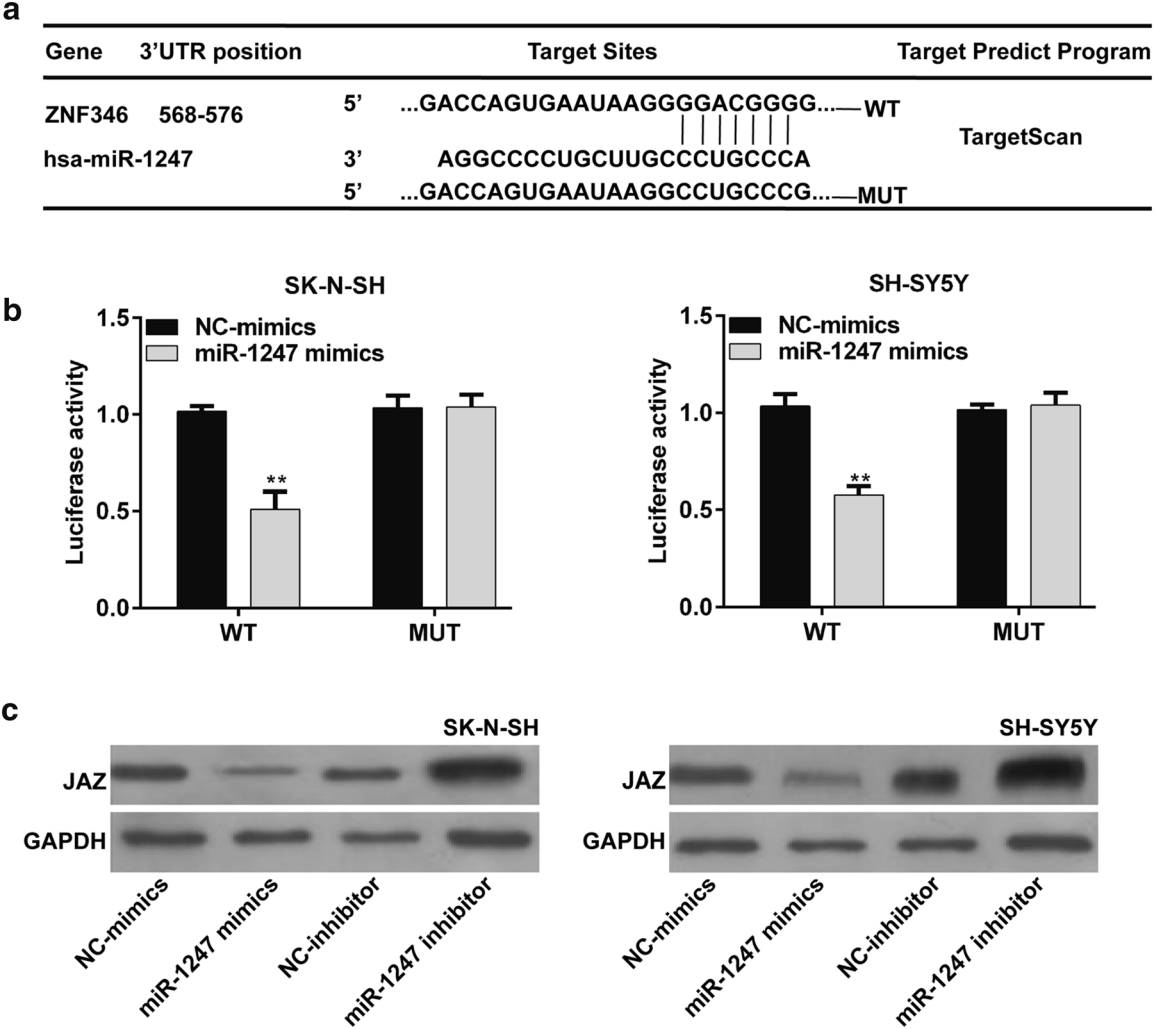
ZNF346 was identified as a target gene of miR-1247 in NB cells. **a** The miR-1247 binding sites in the ZNF346 3′UTR predicted by bioinformatics analysis. **b** Co-transfection of a wild type (WT) or a mutant ZNF346 3′UTR with miR-1247 mimics into SH-SY5Y and SK-N-SH cells. Luciferase activities were measured by Dual-luciferase reporter assay. **c** Western blot showing ZNF346 protein expression levels in SH-SY5Y and SK-N-SH cells after transfection with NC-mimics, miR-1247 mimics, NC-inhibitor and miR-1247 inhibitor, respectively. GAPDH was used as the endogenous control. **p < 0.01 versus NC-mimics

To figure out the molecular mechanisms in which the miR-1247 regulates cell proliferation and apoptosis in NB, we searched for potential target genes of miR-1247 using three tool databases including TargetScan (www.targetscan.org), miRpathDB (https://mpd.bioinf.uni-sb.de/), and microRA.org (http://www.microrna.org/), and found ZNF346 gene was predicted as a putative target for miR-1247, which might be implicated in the pathogenesis of NB (Fig. 5a). To validate this predication, luciferase reporter constructs taking the 3′UTR miR-1247 potential binding site or mutant binding sites of ZNF346 were constructed and co-transfected with miR-1247 mimics or NC-mimics into NB cells. Through performing dual-luciferase reporter assay, we uncovered that only SH-SY5Y and SK-N-SH cells in the WT 3′UTR/miR-1247 mimics group had a remarkably lower luciferase activity than other groups (Fig. 5b, *p* < 0.01). Moreover, over-expression or knockdown of miR-1247 increased and decreased ZNF346 protein expression in both SH-SY5Y and SK-N-SH cells by Western blot analysis (Fig. 5c). The results further demonstrated that miR-1247 directly bound to the 3′UTR of ZNF346 in NB cells.

### Downregulation of ZNF346 induced cell proliferation inhibition and apoptosis in NB cells

**Fig. 6:**
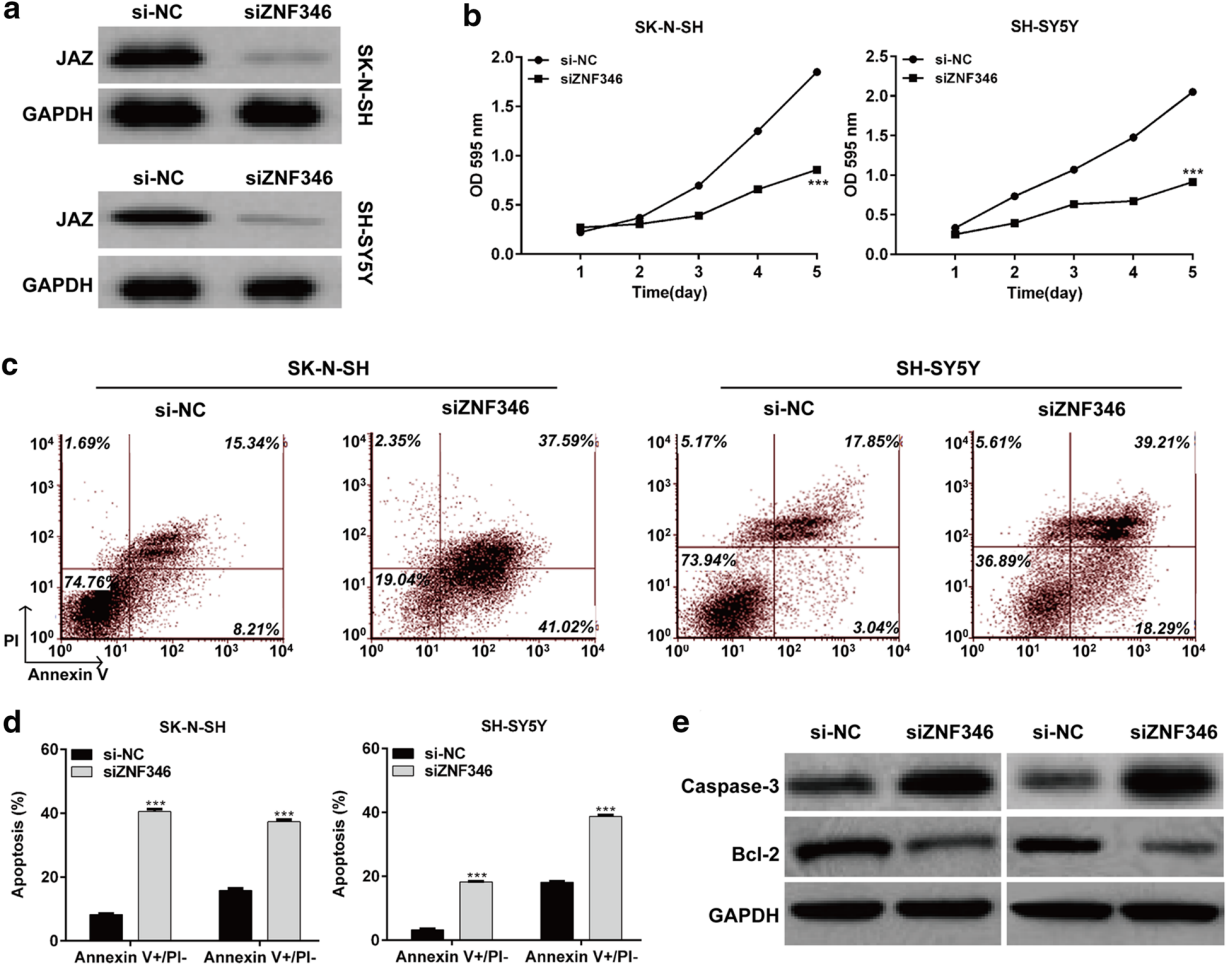
Knockdown of ZNF346 suppresses cell proliferation and promoted apoptosis in NB cells. Cells were respectively transfected with the siZNF346 or si-NC, respectively for 48 h. **a** The expression of ZNF346 protein levels was determined by Western blot analysis in SH-SY5Y and SK-N-SH cells. **b** MTT assay was used to measure cell proliferation in SH-SY5Y and SK-N-SH cells. **c**, **d** Flow cytometry assay was performed to evaluate cell apoptosis in SK-N-SH andSH-SY5Y cells. **e** The protein expression of Caspase 3 and Bcl-2 were analyzed in SK-N-SH and SH-SY5Y cells infected with siZNF346 or si-NC by Western blot. GAPDH was used as the endogenous control. ***p < 0.001 versus si-NC

ZNF346, a member of a new class of Cys-2–His-2 zinc finger proteins, is expressed widely and relatively highly in most organs, including the brain [23]. Thus, we further performed loss-of-function studies to investigate the functional role of ZNF346 in NB. As shown in Fig. 6a, siZNF346 transfection obviously downregulated the expression of protein ZNF346 in both SK-N-SH and SH-SY5Y cells. In functional assays, MTT assay indicated that knockdown of ZNF346 significantly inhibited cell proliferation in NB cells (Fig. 6b, *p* < 0.001). Flow cytometry further demonstrated that knockdown of ZNF346 notably elevated the cell early apoptotic and late apoptotic rate in both SK-N-SH and SH-SY5Y (Fig. 6c, d, *p* < 0.001) cells. Furthermore, we found ZNF346 knockdown obviously elevated the expression of caspase-3, but decreased the expression of Bcl-2 in both SK-N-SH and SH-SY5Y cells (Fig. 6e).

## Discussion

The miRNAs can recognize and directly bind to the 3′UTR sequence motifs of target genes in animals, and negatively regulate the production of the target gene’s protein product, thus affecting malignant cell behavior [24, 25]. The identification of functional roles and the probable mechanisms of miRNAs which abnormally expressed in cancer may supply valuable clues regarding miRNA-therapeutic strategies in oncology [26, 27]. Although miR-1247 is implicated in the progression of several tumor types, and the dysregulation of miR-1247 has also been detected in certain neoplastic tissues, the impacts of miR-1247 on NB and its potential targets have not yet been reported.

In this study, we have obtained the first data about the pathological function of miR-1247 in NB. Our results show that miR-1247 is less abundant in human NB tissues than that in adjacent tissues. Increasing levels of miR-1247 blocked the NB cells proliferation and colony formation and promoted apoptosis, whereas underexpression of miR-1247 had the opposite effect. Our results are similar with previous studies on pancreatic cancer [19], osteosarcoma [28], and NSCLC [21], which reveals that enhanced-expression of miR-1247 causes a decrease in cell viability. Furthermore, potential miR-1247 targets were identified by TargetScan, miRBase, and microRA.org databases. Dual-luciferase reporter assay showed that miR-1247 bound to recognition sites within the 3′UTR of ZNF346 and dramatically downregulated the ZNF346 at the post-translational level in NB cells. These results suggested that ZNF346 was a direct target of miR-1247 in NB. Moreover, the silencing of ZNF346 exerted qualitatively similar effects on cellular behavior as miR-1247 mimics, suggesting a role of ZNF346 in mediating miR-1247 inhibition of cell growth. Interestingly, previous studies reported that miR-1247 targeting neuroplilin 1 and neuroplilin 2 in pancreatic cancer, targeting MAP3K9 in osteosarcoma, and targeting STMN1 in NSCLC, thereby regulated tumor cells proliferation. These investigations revealed that miR1247 probably showed significantly different targets in various types of malignancies.

ZNF346 is a member of the C2H2-type zinc finger proteins that has a neuroprotective role in neurodegenerative disease models [23, 29, 30]. Mallick et al. [23] demonstrated that low ZNF346 expression attenuated neurons survival in vitro. However, ZNF346 expression is not altered in neurons which are primed to undergo apoptosis, implying a critical role for post-translational modification of ZNF346 in neuroprotection [28]. In the present study, overexpression of miR-1247 and silencing of ZNF346 can both stimulate apoptosis. ZNF346 is a direct target of miR-1247 and its protein production was negatively regulated by miR-1247. We supposed that overexpression of miR-1247 in cultured NB cells led to their death though reduced ZNF346 protein levels via post-translational downregulation. Previous studies reported that neurons were also protected by ZNF346 from the following different paradigms, including interaction with SIRT, mut-Htt, and mut-Atx1 toxity. In the future, the concrete mechanism of miR-1247-induced apoptosis mediated by ZNF346 will be focused.

## Conclusions

In summary, low miR-1247 expression in NB tissueswas observed compared with that in adjacent tissues. MiR-1247 suppresses the proliferation and colony formation and induces apoptosis by depressing its target protein ZNF346. This exploration expands our understanding about the regulation of ZNF346 at the post-translational level by miR-1247 in NB and suggests that the dysregulation of miR-1247 emerges a potential pathological mechanism in NB tumorigenesis. Moreover, our investigation may provide valuable clues for developing anti-cancer strategies.
